# Analysis of factors influencing patient delay by patients with pulmonary tuberculosis in Lishui City, Zhejiang Province

**DOI:** 10.1186/s12890-023-02554-w

**Published:** 2023-07-18

**Authors:** Jing Guo, Yin-Ping Feng, Zhong-Da Liu, Shui-Rong Luo, Qian-Yu Wu

**Affiliations:** Department of Tuberculosis, Lishui Hospital of Traditional Chinese Medicine, No. 800 Zhongshan Street, Liandu District, Lishui, 323000 China

**Keywords:** Influencing factor, Lung, Patient delay, Tuberculosis

## Abstract

**Objective:**

The purpose of this study was to collect data on the current state of patient delay by patients with tuberculosis (TB) in Lishui City, Zhejiang Province who were under the care of a TB-designated hospital from 2011 to 2021 and to analyze the factors that contribute to this problem in order to provide a scientific basis for the prevention and control of TB.

**Methods:**

In this observational study, we collected data on patients with pulmonary TB that were reported to the Chinese government's disease prevention and control information system by the Traditional Chinese Medicine Hospital in Lishui City between 2011 and 2021. The data included demographics like age, gender, occupation, household registration, current address, date of symptoms, date of first visit, and etiology results. Multivariate logistic regression analysis was used to analyze the factors influencing patient delay by patients with pulmonary TB.

**Results:**

There were 3,190 cases of pulmonary TB treated in a TB-designated hospital in Lishui City, Zhejiang Province, between 2011 and 2021. Of these, 2,268 involved patient delay, with the delay rate of 71.10% and the median (Q25, Q75) days of patient delay being 36 (25, 72) days. Results of multivariate logistic regression analysis indicated the presence of risk factors-age > 60 years old (OR = 1.367, 95% CI: 1.144 ~ 1.632), pathogen positive (OR = 1.211, 95% CI: 1.033 ~ 1.419), and employed as peasants (OR = 1.353, 95% CI:1.144 ~ 1.601) for patient delay in patients with pulmonary TB. Patients with diabetes mellitus made up 64.94% of the pulmonary TB population, which was lower than the 71.58% of patients without diabetes mellitus (χ^2^ = 4.602, *P* = 0.032). Additionally, the presence of diabetes mellitus may be a protective factor in patient delay in patients with pulmonary TB (OR = 0.641, 95% CI: 0.481 ~ 0.856).

**Conclusion:**

High rates of patient delay, age > 60 years old, a positive etiology, and being employed as peasants are all possible risk factors for pulmonary TB in Lishui City, Zhejiang Province.

## Introduction

Tuberculosis (TB) is a chronic infectious disease that is primarily transmitted through the airways and has a massive global burden [1]. According to the 2021 Tuberculosis Report of the World Health Organization (WHO), China had an estimated 842,000 new patients with TB in 2020, with an incidence rate of 59/100,000 [2]. China had the second-highest number of TB cases out of the 30 countries with the highest TB burden, lower than India (2.59 million). TB is highly contagious and has a high mortality rate [3]. Due to the prevalence of patient delay by TB patients [3], patients carry infectious *Mycobacterium tuberculosis* for an extended period of time and increase the likelihood of TB transmission [4]. In addition, patient delay causes patients to miss the optimal treatment window, resulting in poor treatment outcomes and an increased risk of death [5].

In its early stages, TB often presents with symptoms that are easy to confuse with those of other common febrile diseases. Patients may wait weeks or months before seeking treatment or being diagnosed with active TB because early-stage symptoms are typically not disabling and allow for the continuation of day-to-day activities. Close contacts of the patient are most at risk for infection during this time [6]. Given that factors like poor socioeconomic conditions, limited access or poor quality of services, differences in age, gender, education level, gender, co-infection with the human immunodeficiency virus (HIV), and alcohol and drug abuse all contribute to a delay in diagnosis, it is imperative that these issues be addressed [6].

The time it takes from when a patient first experiences symptoms to when they are diagnosed with TB can be broken down into two categories: patient delays and diagnostic delays. The term "patient delay" describes the length of time that passes between when a person experiences their first sign of illness and when they seek medical attention. The term "diagnosis delay" describes the length of time it takes for a patient to be diagnosed with TB after their initial visit to a medical facility [7]. Time elapsed between the initial consultation and the final diagnosis is typically assumed to be more than two weeks (14 days). The WHO [7] concurs with an Indian study that found the most sensitive screening period was two weeks after the onset of symptoms [8].

China has a high TB burden and is one of the few middle- and high-income countries with a high TB burden. While progress has been made in TB prevention and control in China, the fifth national TB epidemiological sampling survey found that only 47% of patients with TB in China received prompt medical treatment [9]. A study in Nanjing showed that the average delay in diagnosis of TB patients was 50.3 days [10]. A 2018 study estimated that one-third of the people with TB worldwide, remained undiagnosed [11]. In the clinical setting, it can take weeks or months for a patient to receive a diagnosis of tuberculosis. This delay in diagnosis poses a double threat: first, it allows the disease to progress unchecked, missing the window of opportunity for effective treatment; second, it allows TB to spread further in the community, increasing the difficulty of eradicating the disease. Therefore, academics have taken an interest in studies examining the causes of patient delay for people with pulmonary TB. Prevention and control of TB can benefit from a better understanding of the factors that contribute to treatment delays, guidance for policymakers on how to address these issues, prompt identification of active TB cases, and prompt implementation of treatment.

This research aimed to provide a scientific foundation for the prevention and control of TB by collecting data on patients with TB in Lishui City, Zhejiang Province who experienced patient delays, and then analyzing the factors that contributed to those delays.

## Study patients and methods

### Study patients and setting

In this observational study, the case information of patients was obtained from the "TB management information system" subsystem of the "China disease prevention and control information system," based on the pulmonary TB cases diagnosed in Lishui Traditional Chinese Medicine Hospital between January 2011 and December 2021. This study was approved by the Lishui Traditional Chinese Medicine Hospital (Approval number: 2023LW-033).

### Methods

The basic information of patients with pulmonary TB registered and diagnosed in Lishui Hospital of Traditional Chinese Medicine from 2011 to 2021 were collected from the “Tuberculosis Management Information System,” and included gender, age, occupation, household registration, date of symptoms, date of first visit, etiology results, initial treatment/retreatment, complicated with diabetes, and patient source.

### Relevant definitions

Patient delay: The time interval between the onset of symptoms and the first visit that exceeds 2 weeks [12].

Initial treatment: Did not previously receive anti-TB treatment or had been treated for less than one month.

Retreatment: Previously received anti-TB treatment for more than one month.

Etiology result: Results of the *M. tuberculosis* test using sputum smear microscopy, PCR, or culture. A "positive" result indicates the detection of *M. tuberculosis*, while a "negative" result indicates that *M. tuberculosis* was not detected.

Complicated with diabetes: Patients diagnosed with diabetes prior to or simultaneously with the diagnosis of TB.

Patient source: The type of setting that the patient came from.

Transfer: Patients were transferred from non-TB-designated medical institutions or non-TB outpatient clinics.

Referral: Patients were referred by doctors at primary healthcare institutions due to symptoms similar to pulmonary TB or with radiological evidence suggesting suspected pulmonary TB.

### Statistical analysis

Quantitative data that deviate from the normal distribution are described as the median (M (Q25, Q75)), while enumeration data are described as n(%). Statistical analysis was performed using IBM SPSS Statistics 23.0 software. Chi-squared tests or Fisher's exact probability method were utilized for univariate analysis of the factors influencing the patient delay by patients with pulmonary TB, while the logistic regression model was utilized for multivariate analysis. *P* < 0.05 is statistically significant.

## Results

### General conditions of patients

From 2011 to 2021, a total of 3,190 cases of pulmonary TB were recorded at the Traditional Chinese Medicine Hospital in Lishui City. The median time from the onset of symptoms to the first doctor visit was 27 (12, 58) days. The median age was 52 (33, 67) years old. The majority population constituted the Han ethnicity (3095/3190, 97.02%), males (2258/3190, 70.78%), local households (2337/3190, 73.26%), and peasants (1941/3190, 60.85%). Based on the source of patients, 1,287 patients (1287/3190, 40.34%) were transferred, followed by 992 patients (992/3190, 31.10%) who directly visited, and 634 patients (634/3190, 19.87%) who were followed up. In total, 2,991 cases of secondary pulmonary TB, 150 cases of tuberculous pleurisy, 48 cases of hematogenous disseminated pulmonary TB, and 1 case of primary pulmonary TB were diagnosed, with a pathogen positivity rate of 57.37% (1830/3190). In this study, 231 of the 3,190 patients with pulmonary TB had diabetes, and the median visit time for patients with pulmonary TB and diabetes was 25 (9, 66) days less than for those without diabetes (27 (12, 57) days).

### Situation of patients with patient delay

In the Traditional Chinese Medicine Hospital in Lishui City, there were 2,268 cases of patient delay by patients with pulmonary TB from 2011 to 2021, of which 150 cases were complicated by diabetes. The percentage of delayed days was 71.10%, with the median being 36 (25, 72). From 2011 to 2015, these figures demonstrated an upward trend, whereas from 2016 to 2021, they demonstrated a downward trend. The rate of patient delay was higher among peasants than among non-peasants. The patient delay by patients having a local household registration were greater than those with a non-local household registration. The delayed visit rate for people under 60 years old decreased annually, whereas the delayed visit rate for patients with pulmonary TB over 60 years old increased annually (Table 1).

**Table 1:** Patient delay by patients with TB from 2011 to 2021(%)

Years	Total number of delayed doctor visits	Gender		Age		Occupation		Household registration	
		Male	Female	≤ 60 years old	> 60 years old	Peasants	Non-peasants	Local	Non-local
2011	183(183/263,69.58%)	135(73.77)	48(26.23)	111(60.66)	72(39.34)	102(55.74)	81(44.26)	166(90.71)	17(9.29)
2012	216(216/273,79.12%)	142(65.74)	74(34.26)	137(63.43)	79(36.57)	109(50.46)	107(49.54)	198(91.67)	18(8.33)
2013	166(166/191,86.91%)	120(72.29)	46(27.71)	107(64.46)	59(35.54)	107(64.46)	59(35.54)	154(92.77)	12(7.23)
2014	212(212/261,81.23%)	146(68.87)	66(31.13)	149(70.28)	63(29.72)	124(58.49)	88(41.51)	156(73.58)	56(26.42)
2015	178(178/205,86.83%)	117(65.73)	61(34.27)	109(61.24)	69(38.76)	123(69.10)	55(30.90)	131(73.60)	47(26.40)
2016	209(209/266,78.57%)	140(66.99)	69(33.01)	135(64.59)	74(35.41)	120(57.42)	89(42.58)	137(65.55)	72(34.45)
2017	176(176/262,67.18%)	127(72.16)	49(27.84)	108(61.36)	68(38.64)	119(67.61)	57(32.39)	101(57.39)	75(42.61)
2018	194(194/316,61.39%)	124(63.92)	70(36.08)	122(62.89)	72(37.11)	124(63.92)	70(36.08)	111(57.22)	83(42.78)
2019	233(233/363,64.19%)	181(77.68)	52(22.32)	130(55.79)	103(44.21)	168(72.10)	65(27.90)	155(66.52)	78(33.48)
2021	262(262/413,63.44%)	197(75.19)	65(24.81)	131(50.00)	131(50.00)	175(66.79)	87(33.21)	185(70.61)	77(29.39)
2021	239(239/377,63.40%)	174(72.80)	65(27.20)	109(45.61)	130(54.39)	177(74.06)	62(25.94)	177(74.06)	62(25.94)

### Analysis of influencing factors of patient delay

The univariate analysis revealed that among the 3,190 cases of pulmonary TB, the number of patients with patient delay was 2,268, at a rate of 71.10%. Whether the patient was over the age of 60, a peasant, complicated with diabetes mellitus, had a positive etiology, and the source of the patient were statistically correlated with patient delay by patients with pulmonary TB (*P* < 0.05). There was no statistical correlation between gender, initial/re-treatment, household registration, and patient delay among patients with pulmonary TB (*P* > 0.05, Table 2).

**Table 2:** Univariate analysis of factors influencing patient delay by patients with TB

Variable	No patient delay (*n* = 922)		Patient delay (*n* = 2268)		*χ*^2^	*P*
	Cases	Percentage (%)	Cases	Percentage (%)		
Gender						
Male	655	29.01	1603	70.99	0.042	0.838
Female	267	28.65	665	71.35		
Age (years old)						
> 60	281	23.40	920	76.60	28.413	< 0.001
≤ 60	641	32.23	1348	67.77		
Initial/retreatment						
Retreatment	67	26.48	186	73.52	0.784	0.376
Initial treatment	855	29.11	2082	70.89		
Complicated with diabetes						
Yes	81	35.06	150	64.94	4.602	0.032
No	841	28.42	2118	71.58		
Household registration						
Non-local	256	30.01	597	69.99	0.697	0.404
Local	666	28.50	1671	71.50		
Occupation						
Peasants	493	25.40	1448	74.60	29.613	< 0.001
Non-peasants	429	34.35	820	65.65		
Pathogenic results						
Negative	432	31.76	928	68.24	9.449	0.002
Positive	490	26.78	1340	73.22		
Patient source						
Transfer	382	29.68	905	70.32	109.68	< 0.001
Follow up	274	27.62	718	72.38		
Direct visit	133	20.98	501	79.02		
Physical examination	92	64.79	50	35.21		
Referral	41	30.37	94	69.63		

#### Multivariate logistic regression analysis

A multivariate logistic regression analysis was performed on the dependent variable of patient delay (0 = no, 1 = yes) and the independent variables of age > 60, peasant, diabetes, pathogen positivity, and patient source. The results revealed that age > 60 years old, a positive etiology, and peasants were risk factors for patient delay in patients with pulmonary TB, whereas the rate of patient delay in patients with pulmonary TB complicated by diabetes was 64.94%, compared to 71.58% in those without diabetes. Therefore, diabetes may be a protective factor for patient delay in patients with pulmonary TB (Table 3).

**Table 3:** Multivariate logistic regression analysis of factors influencing patient delay by patients with TB

Variable	*β*	*S*_*x*_	Wald^2^	*P*	*OR*	95%*CI*
Age > 60 years old	0.312	0.091	11.898	0.001	1.367	1.144~1.632
Patient source	-0.050	0.036	1.904	0.168	0.951	0.886~1.021
Complicated with diabetes	-0.444	0.147	9.125	0.003	0.641	0.481~0.856
Pathogen positive	0.191	0.081	5.546	0.019	1.211	1.033~1.419
Peasants	0.302	0.086	12.440	< 0.001	1.353	1.144~1.601

## Discussion

This study revealed that the median days of patient delay for patients with pulmonary TB at the Traditional Chinese Medicine Hospital in Lishui City from 2011 to 2021 was 36, which was longer than the 24.6 days in Ethiopia [13]. The patient delay rate was 71.10%, which was higher than 42.60% reported by Chaoyang District of Beijing [14] and 52.9% reported by Huzhou City, Zhejiang Province [12] and 68.6% reported by Hanzhong City, Shaanxi Province [15]. The patient delay rate is high in China and is a matter of concern. The trend increased from 2011 to 2015 and decreased from 2016 to 2021, which may be closely related to the strengthening of TB prevention and control measures in Lishui City [14] such as increasing the training of doctors in medical institutions to improve their ability to identify TB, the disease prevention and control departments or the TB-designated hospitals actively promoting TB prevention and control knowledge and policy promotion on the World TB Day on March 24. These preventative and corrective measures are crucial to reducing patient delay.

The multivariate logistic regression analysis revealed that elderly patients with pulmonary TB > than 60 years old had significantly higher patient delay rates than non-elderly patients. This is consistent with the delayed visit rate of elderly patients with pulmonary TB (61.69%) reported by Xu et al. being significantly higher than that of non-elderly patients (45.19%) during the same period [16]. The delayed visit rate of patients with TB > 60 years old at the Traditional Chinese Medicine Hospital in Lishui City, Zhejiang Province between 2011 and 2021 is 76.60%, which is comparable to the patient delay of 74.3% in elderly patients with TB in Dongguan City, Guangdong Province, between 2009 and 2018 [17]. This may be due to lack of knowledge about TB, low level of family and social support, the high rate of pathogen positivity, the high rate of retreatment, and the occurrence of numerous complications among the elderly. In the promotion of fundamental TB knowledge and early screening, greater emphasis should be placed on the elderly population, as well as on publicity efforts and the publicity effect. Chest X-rays should be routinely utilized among the elderly population to screen for TB.

Due to the prolonged infectiousness of *M. tuberculosis* in patients with a positive etiology, patients frequently undergo a mild to severe development process, and a delayed diagnosis of these patients is more adverse [12]. Based on the results of our study, the delay rate of patients with etiology-positive pulmonary TB visiting the doctor is 73.22% higher than that of patients with etiology-negative pulmonary TB (68.24%), which is a risk factor for patient delay. This is consistent with the patient delay of 81.1% reported by Guan et al. in Dongguan City, Guangdong Province, for elderly patients with pulmonary TB with etiology-positive and simple tuberculous pleurisy, which is significantly higher than the 69.7% for patients who are etiology-negative [17].

Risk of patient delay by patients with TB is high in rural areas [18], and the results indicate that employment as a peasant is a risk factor. The fact that these individuals work long hours, have limited access to health care, and lack knowledge about TB prevention and treatment is likely to delay their doctor visits. In rural and remote areas of China, the delay in diagnosis is severe, with the delay rate for patients with TB working as peasants in Guangzhou City between 2008 and 2018 being 56.10% [19]. These patients typically choose the closest primary medical institution, such as a health center or private clinic for their initial visit, despite the fact that primary medical institutions typically have a low medical level and are unable to diagnose TB, potentially misdiagnosing patients with common diseases such as acute upper respiratory tract infection and pulmonary infection, and delaying treatment of the condition and doctor visits [20]. Consequently, the capacity of primary medical institutions to diagnose TB must be enhanced, and a corresponding intervention is urgently required to aid in the prevention and control of TB.

In this study, 231 of the 3,190 patients with pulmonary TB were diabetic, and their median visit time was 25 days, whereas it was 27 days for those without diabetes. But，the median delay for PTB patients with DM (25 days) was significantly higher than that of PTB patients without DM (6 days) in Beijing [21]. However, the results of our study revealed that 64.94% of patients with diabetes and pulmonary TB experienced patient delay, which was lower than the 71.58% of patients without diabetes and comparable to the lower delay rate of patients with diabetes and pulmonary TB reported by Xiao et al. [22]. This is due to the fact that patients with TB complicated by diabetes may prefer to seek medical care earlier because they may exhibit more pronounced clinical symptoms, a broader range of imaging lesions in the lungs, and a higher positive etiology rate. Therefore, early screening and diagnosis of TB in patients with diabetes, as well as strengthening the comprehensive control and management of TB and diabetes are of immense importance.

The impact of the COVID-19 pandemic on patient delay was not analyzed in this study. It should be noted, however, that the COVID-19 pandemic has been ongoing since the end of 2019, dealing an unprecedented blow to the worldwide effort to eradicate TB. This reverses the improvements in basic TB services and TB burden reduction that have been made in recent years. First, TB detection rates have dropped because of the COVID-19 epidemic. Compared with 2019, the number of new TB cases registered in 2020 fell by as much as 18% from 7.1 million to 5.8 million, the first decline in nearly five years, and the number of registered cases fell back to 2012 levels before rising slightly to 6.4 million in 2021 [23]. Second, the treatment and monitoring of patients with TB have been significantly impacted by the COVID-19 epidemic. The most pressing issue is the delay in diagnosis and treatment for patients with TB, especially those with multidrug-resistant TB. Third, there is no reported clinical connection between COVID-19 and TB, but there are similarities between the symptoms, signs, and chest imaging findings of both diseases. As a result, COVID-19 may impede TB diagnosis. Fourth, although COVID-19 containment measures such as decreased travel and social activities and increased awareness of cough etiquette and mask wearing, may reduce the spread of TB in the community, the risk of TB transmission and infection among family members increases due to more time spent at home and increased delays in patient care [24].

The limitation of this study is that we only included cases from the "Tuberculosis Management Information System," a subsystem of the "China Disease Control and Prevention Information System." Although the sample size was adequate, it may not be generalizable to the entire population. Patients infected with COVID-19 in 2020–2021 who died from other causes before being diagnosed with "tuberculosis" were excluded from the study. Furthermore, while our study examined the factors most likely to influence patient delay, many factors were left out, such as the income of patients and family-related factors.

## Conclusion

In conclusion, more attention should be paid to rural patients, the elderly, and those with positive pathogen tests. On the one hand, TB awareness must be increased, and on the other, the diagnostic and treatment capabilities of medical institutions and medical personnel must be enhanced. In addition, it is recommended that primary medical institutions include TB diagnosis techniques such as chest X-ray, sputum smear for acid-fast bacilli, and PPD test in the annual physical examination of the elderly in rural areas, to improve the accessibility of TB prevention and treatment services, identify patients in a timely manner, and carry out early intervention to shorten the delay in diagnosis and treatment of patients with TB and increase the treatment success rate. In recent years, a large amount of research has been conducted on patient delay, and it has been determined that the factors leading to the delay are diverse. However, relatively little research has been conducted on intervention measures for delayed diagnosis. Based on an understanding of the specific reasons for the delay in different populations in different regions, it is recommended that in the future, targeted intervention should be carried out to reduce the occurrence of diagnostic delay, strengthen the multi-sectoral cooperation mechanism, effectively prevent, control, and manage TB, and provide the foundation for the promotion of TB prevention and treatment in China.

## Data Availability

All data generated or analysed during this study are included in this article. Further enquiries can be directed to the corresponding author.

## References

[1] Zhang H, Zhao YL (2022). Strengthen multi-sectoral cooperation mechanism to further promote the tuberculosis prevention and control in China. Chin J Antituberculosis.

[2] World Health Organization. Global tuberculosis report 2021. Geneva: World Health Organization; 2021.

[3] Bello S, Afolabi RF, Ajayi DT (2019). Empirical evidence of delays in diagnosis and treatment of pulmonary tuberculosis: systematic review and meta-regression analysis[J]. BMC Public Health.

[4] Mhalu G, Weiss MG, Hella J (2019). Explaining patient delay in healthcare seeking and loss to diagnostic follow-up among patients with presumptive tuberculosis in Tanzania: a mixed-methods study. BMC Health Serv Res.

[5] Teo AKJ, Singh SR, Prem K (2019). Delayed diagnosis and treatment of pulmonary tuberculosis in high-burden countries: a systematic review protocol. BMJ Open.

[6] Getnet F, Demissie M, Assefa N, Mengistie B, Worku A (2017). Delay in diagnosis of pulmonary tuberculosis in low-and middle-income settings: systematic review and meta-analysis. BMC Pulm Med.

[7] World Health Organization (2011). Early detection of tuberculosis: an overview of approaches, guideIines and tools.

[8] Thomas A, Chandrasekaran V, Joseph P (2008). 1ncreased yield of smear positive pulmonary TB cases by screening patients with>or =2 weeks cough, compared to>or=3 weeks and adequacy of 2 sputum smear examinations for diagnosis. Indian J Tuberc.

[9] Wang Y (2011). The fifth national tuberculosis epidemiological survey.

[10] Martinez L, Xu L, Chen C (2017). Delays and Pathways to Final Tuberculosis Diagnosis in Patients from a Referral Hospital in Urban China. Am J Trop Med Hyg.

[11] World Health Organization (2019). Global tuberculosis report 2019.

[12] Fu LJ, Wang YS, Zhu WL (2021). Consultation delay and influencing factors among pulmonary tuberculosis patients in Huzhou City from 2008 to 2018. Chin J Dis Control Prev.

[13] Alene M, Assemie MA, Yismaw L (2020). Patient delay in the diagnosis of tuberculosis in Ethiopia: a systematic review and Meta-analysis. BMC Infect Dis.

[14] Yu N, Wei YF (2022). Medical care seeking delay and related factors in pulmonary tuberculosis patients in Chaoyang district, Beijing, 2014–2020. Dis Surveill.

[15] Wei JJ, Zeng LX (2018). Status and associated factors analysis of health seeking delay among pulmonary tuberculosis patients in Hanzhong City (2014–2017). J Public Health Prevent Med.

[16] Xu J, Luo P, He XX (2021). Analysis of characteristics and diagnosis delay of 629 elderly tuberculosis patients. J Tuberc Lung Dis.

[17] Guan FY, Chen ZH, Li WH (2021). Characteristics analysis of diagnosis delay among elderly tuberculosis patients in Dongguan City from 2009 to 2018. J Tuberc Lung Dis.

[18] Tong Y, Guan X, Hou S (2018). Determinants of Health Care-Seeking Delay among Tuberculosis Patients in Rural Area of Central China. Int J Environ Res Public Health.

[19] Shen HC, Du YH, Zhang GC (2020). Influencing factors of pulmonary tuberculosis patient delay in Guangzhou, 2008-2018. Chin J Antituberculosis.

[20] Yang Q, Tong Y, Yin X (2020). Delays in care seeking, diagnosis and treatment of patients with pulmonary tuberculosis in Hubei. China Int Health.

[21] Chen HG, Liu M, Jiang SW (2014). Impact of diabetes on diagnostic delay for pulmonary tuberculosis in Beijing. Int J Tuberc Lung Dis.

[22] Xiao W, Huang D, Li S (2021). Delayed diagnosis of tuberculosis in patients with diabetes mellitus co-morbidity and its associated factors in Zhejiang Province, China. BMC Infect Dis.

[23] World Health Organization (2022). Global tuberculosis report 2022.

[24] Fox GJ, Nhung NV, Sy DN (2018). Household-Contact Inves-tigation for Detection of Tuberculosis in Vietnam. N Engl J Med.

